# Effects of glucagon-like peptide-1 receptor agonists (GLP-1RAs) on podocytes, inflammation, and oxidative stress in patients with diabetic nephropathy (DN)

**DOI:** 10.12669/pjms.38.5.4719

**Published:** 2022

**Authors:** Jie Liu, Shanshan Guo, Hui Li, Xu-ying Liu

**Affiliations:** 1 Jie Liu, Department of Endocrinology, Affiliated Hospital of Hebei University, Baoding 071000, Hebei, China; 2 Shanshan Guo, Department of Nephrology, Affiliated Hospital of Hebei University, Baoding 071000, Hebei, China; 3 Hui Li, Department of Neurology, Affiliated Hospital of Hebei University, Baoding 071000, Hebei, China; 4 Xu-ying Liu Department of Endocrinology, Affiliated Hospital of Hebei University, Baoding 071000, Hebei, China

**Keywords:** Diabetic nephropathy (DN), Liraglutide, Podocalyxin (PCX), Nephrin

## Abstract

**Objectives:**

To investigate the effects of a glucagon-like peptide-1 receptor agonist (GLP-1RA) liraglutide on podocytes, inflammation, and oxidative stress in patients with diabetic nephropathy (DN).

**Methods:**

Eighty-four DN patients treated by the department of endocrinology of the Affiliated Hospital of Hebei University during December 2017 and March 2019 were randomly assigned to a control group and a treatment group (n=42, respectively), with the control group prescribed with conventional DN medications and the treatment group receiving liraglutide treatment in addition to the conventional therapy. The course of treatment lasted for 12 weeks. hemoglobin A1c (HbA1C), body mass index (BMI), total cholesterol (TC), triglyceride (TG), urinary albumin excretion rate (UAER), urine podocalyxin (PCX), urine nephrin, as well as inflammation and oxidative stress markers such as tumor necrosis factor α (TNF-α), monocyte chemotactic protein-1 (MCP-1), glutathione peroxidase (GSH-Px), and malondialdehyde (MDA) were measured pre- and post-treatment for intergroup comparison.

**Results:**

After 12 weeks of treatment, HbA1C, BMI, TC, and TG in both groups were reduced in comparison with the pre-treatment levels, with the levels in the treatment group lower than in the control group (*p*<0.05); reduced levels of UAER, PCX, and nephrin were detected in the two groups, with the treatment group exhibiting a significant reduction in these markers compared with the control group (*p*<0.05); the 12-week treatment led to decreases in the TNF-α, MCP-1, and MDA levels in both groups, with the decline in the treatment group exceeding that in the control group, whereas both groups had an increased level of GSH-Px, with the level in the treatment group higher than that in the control group, and the differences were statistically significant (*p*<0.05, respectively).

**Conclusions:**

Liraglutide protects the kidneys and improves DN by inhibiting inflammation and oxidative stress, reducing urinary albumin excretion and podocyte damage and supporting renal function in addition to its hypoglycemic properties.

## INTRODUCTION

Diabetic nephropathy (DN) is one of the most common complications of Type-2 diabetes, which has become a leading cause of death in patients with end-stage renal disease^1^,^2^ as well as in those with diabetes.^3^,^4^ It is suggested that DN is affected by numerous factors such as glomerular hemodynamic alterations, activated renin-angiotensin-aldosterone system (RAAS), lipid metabolism disorders, and increased cytokines, which underpins the need for new therapeutic approaches to prevent and slow the progress of DN. The glucagon-like peptide-1 receptor agonist (GLP-1RA) liraglutide is a novel hypoglycemic agent. Studies indicate that GLP-1RAs have renal protective properties, independent of the hypoglycemic effects.^5^ This study used liraglutide as an add-on to the conventional treatment for DN patients to investigate the renal protective mechanism of liraglutide in addition to its hypoglycemic properties.

## METHODS

In this study, 84 DN patients treated by the department of endocrinology of the Affiliated Hospital of Hebei University between December 2017 and March 2019 were divided into a control group and a treatment group (n=42, respectively) with a random number table.

### Ethical approval:

The study was approved on May 18, 2021 by the Institutional Ethics Committee of Affiliated Hospital of Hebei University, and written informed consent was obtained from all participants

### Inclusion criteria:

- At the age between 18 and 65;
- GLP-1RA naive, and meeting the standard of DN diagnosis;^6^
- Voluntary participation with informed consent.

### Exclusion criteria:

- Non-diabetes induced renal conditions;
- Presence of serious acute/chronic complications of diabetes;
- Pregnant or breastfeeding women;
- Severe mental disorders;
- Relevant contraindications and allergies.

The control group consisted of 22 male and 20 female patients, with the mean age of (45.38 ±10.34) years (range: 36-63) and the course of diabetes of (9.37 ±5.81) years. The treatment group had 21 male and 21 female patients, with the mean age of (46.89 ±11.26) years (range: 38-65) and the course of diabetes of (10.04 ±6.12) years. There were no significant differences between the two groups in age, course of disease, and sex (*p*>0.05).

### Treatment methods:

Conventional treatment was used in the control group, including diabetes diet, exercise education, oral antidiabetic drug or insulin, blood pressure (BP) control, and treatment of lipid metabolism disorders. In the treatment group, liraglutide injection was used in combination with conventional treatment, with an initial dose of 0.6 mg subcutaneously once a day for one week; during the second week, the dose of the once-daily treatment was increased to 1.2 mg, followed by an additional increment to the maximum daily dose of 1.8 mg, if needed. Both groups were under close observation for 12 weeks.

### Outcome Measures:

Blood glucose (BG), body mass index (BMI), and blood fat (BF), as well as kidney function, inflammation, and oxidative stress markers, were monitored in both groups. Body weight was measured to calculate the BMI. Hemoglobin A1c (HbA1C), total cholesterol (TC), triglyceride (TG), and urinary albumin excretion rate (UAER) were tested by the hospital’s laboratory department.Urine Podocalyxin (PCX), urine nephrin, tumor necrosis factor α (TNF-α), monocyte chemotactic protein-1 (MCP-1), malondialdehyde (MDA), glutathione peroxidase (GSH-Px) were determined using the ELISA.

### Staistical Analysis:

The software SPSS 20.0 was used for data processing. Measurement data conforming to normal distribution were expressed by (x̄ ±s), and intergroup comparison was examined by the t-test; enumeration data were represented by frequency or percentage (%), and intergroup comparison was examined by the χ2 test. Results were considered significant if *p*<0.05.

## RESULTS

Following the 12-week treatment, HbA1c, TC, TG, and BMI in both groups were reduced in comparison with the pre-treatment levels, and there were significantly greater decreases in the treatment group than in the control group (*p*<0.05, respectively). Table-I.

**Table I:** Pre- and post-treatment HbA1c, BMI, TC, and TG variations in the control and treatment groups (χ¯±s, n =42, respectively).

Group	Period	HbA1c (%)	BMI (kg/m^2^)	TC (mmol/L)	TG (mmol/L)
Control	Pre-treatment	9.81±0.96	27.42±2.21	7.02±0.89	3.89±0.75
	Post-treatment	7.16±0.62^Δ^	24.10±3.14^Δ^	5.67±0.81^Δ^	2.78±0.58^Δ^
Treatment	Pre-treatment	9.98±0.87	27.83±2.71	7.13±0.91	4.02±0.74
	Post-treatment	6.45±0.51^*Δ^	22.49±3.35^*Δ^	4.35±0.62^*Δ^	1.75±0.44^*Δ^

Δ *p*<0.05 in comparison between the pre- and post-treatment levels;

* *p*<0.05 in comparison between the treatment group and the control group.

Reduced levels of UAER, PCX, and nephrin were observed in both groups after treatment, with the treatment group exhibiting a sharper decline compared with the control group, and the differences were statistically significant (*p*<0.05). Table-II. Intergroup comparison of TNF-α, MCP-1, GSH-Px, and MDA levels before and after treatment

**Table II:** Pre- and post-treatment UAER, PCX, and nephrin variations in the control and treatment groups (χ¯±s, n =42, respectively).

Group	Period	UAER (μg/min)	PCX (ng/ml)	nephrin (ng/ml)
Control	Pre-treatment	288.62±32.24	21.21±4.12	76.46±13.43
	Post-treatment	182.37±28.56^Δ^	15.15±2.36^Δ^	50.11±11.31^Δ^
Treatment	Pre-treatment	282.47±31.86	22.02±3.27	78.23±13.51
	Post-treatment	141.32±27.62^*Δ^	10.23±2.05^*Δ^	36.25±10.37^*Δ^

Δ *p*<0.05 in comparison between the pre- and post-treatment levels;

* *p*<0.05 in comparison between the treatment group and the control group.

The TNF-α, MCP-1, and MDA levels in both groups were reduced after treatment, and the decreases in the treatment group were larger than in the control group; the GSH-Px levels in both groups were increased in comparison with the pre-treatment levels, and the post-treatment GSH-Px level in the treatment group was higher than in the control group; the differences had statistical significance (*p*<0.05, respectively) Table-III.

**Table III:** Pre- and post-treatment TNF-α, MCP-1, MDA, and GSH-Px variations in the control and treatment groups (χ¯±s, n =42, respectively)

Group	Period	TNF-α (ng/L)	MCP-1 (ng/L)	MDA (umol/L)	GSH-Px (U/L)
Control	Pre-treatment	21.12±4.41	19.45±1.67	78.48±4.12	115.26±15.53
	Post-treatment	16.13±3.16^Δ^	15.34±2.14^Δ^	42.31±3.62^Δ^	166.42±20.34^Δ^
Treatment	Pre-treatment	21.76±4.23	13.89±4.15	76.92±4.86	122.38±14.97
	Post-treatment	12.45±2.77^*Δ^	5.58±1.90^*Δ^	24.62±2.52^*Δ^	195.23±22.23^*Δ^

Δ *p*<0.05 in comparison between the pre- and post-treatment levels;

* *p*<0.05 in comparison between the treatment group and the control group.

## DISCUSSION

Diabetic nephropathy (DN) has a particularly complex pathogenesis. In addition to glomerular hemodynamic alterations, lipid metabolism disorders, and activated RAAS, chronic inflammation and oxidative stress are also involved in the development and progression of the disease.^7^,^8^ The pro-inflammatory cytokine TNF-α can induce massive production of protein hydrolysates and superoxides, resulting in enhanced white blood cell formation, increased permeability of the glomerular basement membrane (GBM), and exacerbated renal injury.^9^ Monocyte chemotactic protein-1 (MCP-1), one of the most important members of the pro-inflammatory chemokine family, stimulates extracellular matrix (ECM) accumulation in the glomerular mesangium and promotes GBM thickening and glomerulosclerosis through activation and chemotaxis of mononuclear macrophages.^10^ Once DN occurs, reactive oxygen species increase in the body, leading to elevated oxidative stress,^11^ glomerular injury, renal matrix remodeling, and tubulointerstitial fibrosis.^12^ Malondialdehyde (MDA) is a peroxidation intermediate that reflects the degree of oxidative damage as a lipid peroxidation marker. A higher MDA level indicates greater cell damage.^13^ Glutathione peroxidase (GSH-Px) is an important intrinsic antioxidant enzyme that eliminates harmful oxides and mitigates cell damage induced by excessive oxidative stress.^14^

Liraglutide is a glucagon-like peptide-1 receptor agonist (GLP-1RA). GLP-1RAs have been shown to protect kidney function independent of the hypoglycemic effect. Studies indicate that GLP-1RAs affect the RAAS, nervous system, and atrial natriuretic peptide to reduce inflammatory response, inhibit oxidative stress, modulate advanced glycation endoproducts (AGEs) due to their renal protective roles.^15^-^17^ In DN patients, glycolipid metabolism, biochemical variations, and hemodynamic alterations may induce inflammatory response. A range of inflammatory cells and factors play an important role in promoting the development and progression of DN. Liraglutide is shown to inhibit inflammatory response and delay DN progression in diabetic rat models by reducing the expression of ERK1/2 and JNK phosphorylation and downregulating the nuclear NF-кB expression.^18^ In the diabetic setting, increased mitochondrial ROS (mROS) can bring structural and functional damage to the kidneys, resulting in the development and progression of DN. It has been found that the cAMP-PKA pathway can be stimulated by GLP-1RAs, which reduces ROS production by the kidneys in DN patients, suggesting the renal protective role of GLP-1RAs in preventing oxidative damage.^19^ The results of this study showed that compared with the pre-treatment levels, TNF-α, MCP-1, and MDA in both groups were reduced after 12 weeks of treatment, whereas the GSH-Px levels were elevated, with the treatment group displaying greater variations than the control group, and the differences were statistically significant (*p*<0.05, respectively). This indicates that liraglutide is protective of the kidneys by inhibiting inflammation and oxidative stress, independent of its hypoglycemic properties.

Glomerular podocytes are highly differentiated cells that support glomerular filtration barrier integrity. Functional impairment or decrease in glomerular podocytes is suggested to induce elevated urinary albumin excretion and glomerulosclerosis.^20^ Complexes in podocyte foot processes (FPs) are composed of various proteins such as PCX and nephrin to maintain morphological and functional characteristics of podocytes and form a glomerular filtration barrier. Podocalyxin (PCX) is the central component of glycocalyx that imparts a high degree of negative charge on the podocyte surface to maintain podocyte architecture and modify the structure of slit diaphragms, suggesting its critical role in supporting the structural and functional integrity of the glomerular filtration barrier.^21^ Evidence has shown that urine PCX in DN patients is higher than in healthy individuals, suggesting the use of increased urine PCX as a marker for podocyte damage in early DN and target of DN treatment.^22^ Nephrin is a secretory protein in the slit diaphragm that reduces podocyte apoptosis and supports the glomerular filtration barrier by modulating the podocyte architecture, as well as relevant signaling pathways, and interacting with Podocin and CD2AP.^23^ Inflammation and oxidative stress are reported to have contributory effects on the expression alterations of nephrin on the podocyte surface, which interferes with the structural stability of podocytes and results in glomerular filtration dysfunction and albuminuria.^24^ In this study, the urine levels of PCX and nephrin in DN patients were both increased before treatment. Following 12 weeks of treatment, the urine levels of PCX and nephrin were reduced to lower than the pre-treatment conditions (*p*<0.05), with the treatment group showing significant decreases compared with the control group (*p*<0.05). This suggests that liraglutide protects kidney function by reducing podocyte damage, maintaining glomerular infiltration barrier integrity and inhibiting urinary albumin excretion.

### Limitations of this study:

The sample size of this study is not large enough. If the sample size can be further expanded, the conclusion may be more convincing. In addition, further studies with large-scale standard treatment are needed to confirm the curative effect, safety, optimum dose, and duration of thymosin treatment.

## CONCLUSION

Although DN has a highly complex pathogenesis, the GLP-1RA liraglutide manages to fulfill its renal protective effects by inhibiting inflammation and oxidative stress, supporting formation and maintenance of podocytes and reducing urinary albumin excretion, providing a novel theoretical basis for DN treatment with favorable clinical efficacy and extensive social benefits.

### Authors’ Contributions:

**JL & SSG:** Designed this study and prepared this manuscript, and are responsible and accountable for the accuracy or integrity of the work.

**HL:** Collected and analyzed clinical data.

**XYL:** Significantly revised this manuscript.
